# Pharmacokinetics and biosafety evaluation of a veterinary drug florfenicol in rainbow trout, *Oncorhynchus mykiss* (Walbaum 1792) as a model cultivable fish species in temperate water

**DOI:** 10.3389/fphar.2023.1033170

**Published:** 2023-01-23

**Authors:** Sumanta Kumar Mallik, Neetu Shahi, Richa Pathak, Krishna Kala, Prasanna Kumar Patil, Bhupendra Singh, Rajisha Ravindran, Nanitha Krishna, Pramod Kumar Pandey

**Affiliations:** ^1^ Diagnostic Bacteriology Laboratory, Fish Health Section, ICAR-Directorate of Coldwater Fisheries Research (ICAR-DCFR), Nainital, Uttarakhand; ^2^ Aquatic Animal Health and Environment Division, ICAR- Central Institute of Brackishwater Aquaculture (ICAR-CIBA), Tamil Nadu, India; ^3^ Quality Assurance and Management, ICAR- Central Institute of Fisheries Technology (ICAR-CIFT), Kochi, India

**Keywords:** rainbow trout, florfenicol, florfenicol amine, distribution, elimination, biosafety

## Abstract

In two experimental trials; florfenicol pharmacokinetics following a single dose oral administration at 15 mg kg^−1^ fish body weight and biosafety through extended medicated feeding were studied in the rainbow trout, *Oncorhynchus mykiss*. The pharmacokinetic trial was conducted for 5 days, whereas the biosafety experiment lasted for a 30-day safety margin followed by a 20-day residual period analysis at 3, 5 and 10 times greater than the therapeutic dose 10 mg kg^−1^ biomass day^−1^. *C*_max_ µg kg^−1^ calculated for florfenicol were found to be 5,360 in intestine, 2,890 in gill, 2,250 in kidney, 973 in liver and 273 in plasma, obtained at *T*_max_ of 16 h. Intestine had utmost area under the concentration–time curve _(tissue/plasma)_ of 13.83 h μg kg^−1^ and a prolonged half life (t_1/2ß_) of 28.62 h. The highest apparent metabolic rate value in the kidney (0.327) showed a high level of biotransformation of florfenicol to its metabolite florfenicol amine. The apparent distribution rate of florfenicol amine in muscle, in comparison to the parent drug florfenicol, indicated elimination of the medication mostly in the form of florfenicol amine with t_1/2_ of 16.75 h. The biosafety of florfenicol orally administered to rainbow trout recorded effective feed consumption, physiological responses, drug tolerance and significantly low drug concentrations in muscle of rainbow trout, thus its usage at 10 mg kg^−1^ fish body weight is recommended. In the study, the rapid absorption, greater bioavailability, enhanced dispersion, slower elimination and biosafety of the drug form a significant basis for the florfenicol and its metabolite florfenicol amine as a useful antibacterial agent in aquaculture.

## 1 Introduction

The Food and Agriculture Organization (FAO) has approved few antibiotics for their use in aquaculture (Serrano, 2005) and florfenicol is one of them. Florfenicol, (C_12_H_14_Cl_2_FNO_4_S) is considered as a synthetic veterinary antimicrobial agent with similar chemical structure and antibacterial activity to thiamphenicol. It is chemically a sulfone, a secondary alcohol, an organofluorine compound, an organochlorine compound and a secondary carboxamide. The mechanism of florfenicol action includes inhibition of synthesis of protein by binding to the bacterial 50S ribosomal subunit at peptidyl transferase stage that prevents protein formation. Moreover, the diffusion process facilitates the drug’s ease penetration in the bacterial cells and lipophilic nature of florfenicol helps to cross some anatomic barriers (Lunestad and Samuelsen 2008; Sutili and Gressler 2021). It is primarily bacteriostatic, and has demonstrated broad spectrum antibacterial activity against Gram-negative bacilli, Gram-positive cocci, and several other pathogenic bacteria in fish.

In aquaculture, florfenicol is widely used against *Aeromonas hydrophila, A. salmonicida, Vibrio anguillarum, Edwardsiella tarda, Edwardsiella ictaluri, Flavobacterium psychrophilum* and *F. columnare*. Besides the therapeutic value, it is essential to know the pharmacokinetics and biosafety of the drug for its effective usages in fish species of commercial importance. The pharmacokinetics profile of florfenicol have been described in Atlantic salmons (Martinsen et al., 1993; Horsberg et al., 1996), Korean catfish (*Silurusasotus*) (Park et al., 2006), koi carp (*Cyprinus carpio*) (Yanong et al., 2005), cod (*Gadus morhua*) (Samuelsen et al., 2003), red pacu (*Piaractus brachypomus*) (Lewbart, 2005), tilapia (*Oreochromis* spp.) (Feng et al., 2008; Feng et al., 2008; Gaikowski et al., 2010), crucian carp (*Caracius auratus cuvieri*) (Sun et al., 2010), olive flounder (*Paralichthys olivaceus*) (Lim et al., 2010), channel catfish (*Ictalurus punctatus*) (Gaunt et al., 2012), yellow catfish (*Pelteobalgrus fulvidraco*) (Yang et al., 2013) and rainbow trout (*Oncorhynchus mykiss*) (Pourmolaie et al., 2015). Florfenicol is typically applied through fish feed (either before pelleting or coated onto pellets) and administered at a dose of 10 mg kg^−1^ body weight for 10 consecutive days in aquaculture to treat bacterial diseases in Atlantic salmon (Martinsen et al., 1993; Horsberg et al., 1996). The findings reveal that florfenicol is rapidly absorbed and widely disseminated in Atlantic salmon, cod, and Korean catfish, with 91%–99% bioavailability. The pharmacokinetics of florfenicol in fish has been investigated in plasma, muscle, and liver. The skin, gill, intestine, bile, and kidney were the subjects of only a few research findings (Feng et al., 2016). In recent years, the importance has been given to develop “optimal antibiotic therapy” by standardizing dosing regimens of antimicrobials, with consideration of bacterial eradication and control of microbial infection to resolve issue like antimicrobial resistance in global fisheries and aquaculture. Pharmacokinetic knowledge and biosafety of the antibiotic in fish are very important to establish the appropriate dosing, improving treatment efficacy and minimizing adverse impact on environment.

The freshwater fish species, rainbow trout, *O. mykiss* (Walbaum 1792) is globally cultivated and considered as an economically important candidate species in aquaculture. However, disease incidences and health challenges associated with various microbial infections, posed a major threat to trout farming and led to increased usages of antibiotics as therapeutic and prophylactic measures without understanding pharmacokinetics, biosafety and treatment effects pertaining to the different culture conditions. It is a fact that the pharmacokinetics parameters of the antibiotic directly affect its therapeutic effect/outcome. Though, there is no dearth of data on pharmacokinetics in fish that may help to set optimum dosing regimens for the antibiotic and reducing environmental impact, the data on pharmacokinetic are notably varied among fish species due to variation in antibacterial property of the microbial agents. Moreover, in some cases, the elevated doses are constantly required to achieve the therapeutic values. So, the compressive studies on pharmacokinetics of the antibiotic must be conducted in different fish species of economic importance in aquaculture, considering their culture conditions. In the return, this may help to understand the intricacy in the drug distribution, absorption and elimination in the fish body. Besides pharmacokinetics, the biosafety of florfenicol in fish is also a major issue as it has a deleterious effect on fish body. It is reported that antibiotic usage may affect immunological responses/systems of the fish (Lunden and Bylund, 2000; Guardiola et al., 2012). The long-term exposure may cause nephrotoxicity and liver damage (Horsberg and Berge, 1986; Hentschel et al., 2005). In addition to that, injudicious usage of antibiotic may cause residual problem in fish tissue and fish products (Samanidou and Evaggelopoulou, 2007), which may become hazardous to fish eaters. In the present study, the disposition of florfenicol and its major metabolite florfenicol amine is investigated in seven different tissues; gill, muscle, liver, kidney, plasma, skin and intestine of rainbow trout after a single dose oral administration through feed reared at 17°C. In another trial, the biosafety of florfenicol coated feed orally administered to rainbow trout was evaluated at 10, 30, 50, and 100 mg kg^−1^ fish biomass day^−1^ for 30 consecutive days, three times higher the suggested 10-day therapeutic dosage, at 19.5°C. The safety issue of the florfenicol is studied with respect to its effect on the feed intake, physiological responses, mortality, histological alterations and the residue clearance in rainbow trout.

## 2 Materials and methods

### 2.1 Chemical

Florfenicol (Cat. No. F1427-500MG) was procured from Sigma Aldrich, USA for the drug analysis in the present pharmacokinetic and biosafety study.

### 2.2 Experimental fish

Healthy rainbow trout juveniles (average weight 138.5 ± 0.015 g and average length 21.5 ± 0.021 cm) were obtained from the re-circulatory aquaculture system (RAS), ICAR-DCFR, Bhimtal and were distributed into circular fibreglass reinforced plastic (FRP) tanks of 1 ton capacity each as treatment and control in triplicates in the wet laboratory. Each experimental tank was stocked with 30 nos. of rainbow trout juvenile. The test fish were acclimatized for the 15 days and fed with a drug-free diet at 2.0% of the body weight (BW) prior to the trial. To improve the feed acceptance, the fish were starved for 12 h before receiving the medicated feed.

### 2.3 Medicated feed preparation

The fish were fed the pelleted commercial diet (NUTRILA, feed code 41945183), manufactured by Growel feeds Pvt. Ltd. R. S. No. 57 and 58, Sriharipurum Panchayat, Krishna district -521329, Andhra Pradesh, India. It was a high quality extruded feed for rainbow trout with crude protein (min 45%), crude fat (min 18%), crude fiber (max 2%) and moisture (max 12%). The important ingredients of the experimental diet were fish meal, soybean meal, wheat products, rice products, fish oil, vegetable oil, soy lecithin, amino acids, vitamins and minerals. The required quantity of florfenicol powder was weighed as per the experimental dose, 15 mg kg^−1^ fish BW. It was mixed with 5% refined vegetable oil and then applied to the pelleted feed. The mixture (vegetable oil, antibiotic and feed) was coated to the feed homogenously by continuous stirring. The control feed was prepared as mentioned above, but without adding the test antibiotic. The control and florfenicol feeds were air dried for a day after proper mixing and stored separately at 4 C in airtight plastic containers.

### 2.4 Physico-chemical parameters

By standard methods for examination of water and wastewater (APHA, 2005), the water quality parameters measured during the experimental trial were; pH (7.5), dissolved oxygen (7.8 mg L^−1^), alkalinity (80 mg L^−1^), water temperature (17.0°C), calcium hardness (80 mg L^−1^), ammonia (0.02 mg L^−1^), nitrite (0.1 mg L^−1^–10 mg L^−1^) and nitrate (10 mg L^−1^). The water quality parameters were in the optimum range to avoid any stressful conditions imparted to the test fish.

### 2.5 Florfenicol administration and sample collection

Florfenicol at a single dose (15 mg kg^−1^ fish body) orally administered to the rainbow trout. Different tissue samples; plasma, liver, kidney, intestine, muscle, gill and skin were collected at different sampling points, i. e. 0, 2, 3, 4, 6, 8, 12, 16, 24, 32, 48, 64, 96, and 128 h post administration of the drug through feed. From each tank, 3 nos. of rainbow trout were collected, pooled together and processed for the collection of above said tissue samples. The blood sample was drawn from the caudal vein with a heparinised 1 mL syringe and centrifuged immediately at 1,000 g for 10 min for the separation of plasma. For florfenicol (FFC) and Florfenicol amine (FFA) LC-MS/MS analysis, all the samples were immediately frozen and stored at −20 C.

### 2.6 Sample preparation for florfenicol and Florfenicol amine LC-MS/MS analysis

Two Gram (2 g) of chopped and homogenised fish sample was weighed into a 50 mL centrifuge tube. 10 mL of ethyl acetate with 2% ammonium hydroxide was used to extract the samples. The sample was vortexed for 2 min to improve analyte extraction. After vortex, the samples were kept in agitation for 20 min, followed by centrifugation at 4,000 rpm at 4°C for 10 min. The supernatants were transferred to a clean tube and evaporated until dry under a gentle flow of nitrogen. Dry residues were subjected to fat removal using 2 mL of hexane, followed by liquid-liquid extraction using 2 mL of water: acetonitrile (50:50,v/v). An aliquot of 1 mL of the bottom layer was transferred to an HPLC vial after soft agitation and analysed in an LC-MS/MS system.

### 2.7 Instrumentation and analytical conditions

An Exion LC system was used in the combination with an AB Sciex 4000 QTRAP triple quadrupole mass spectrometer. The chromatographic separation was performed on a Kinetex C18 column (2.6 m, 150 mm 2.1 mm). The gradient of elution used water containing 10 mM ammonium acetate with 0.1% formic acid (A) and ACN (B) as the mobile phase. The flow rate was 500 μL min^−1^. The gradient began with 15% B and increased to 85% in 2.5 min, then held for 2 min before reducing to 15% in 10 min.

### 2.8 Pharmacokinetic analysis

The non-compartmental pharmacokinetic model based on statistical moment theory was used to determine the pharmacokinetic parameters of florfenicol and florfenicol amine. The trapezoidal method was used to calculate the area under the concentration–time curve (AUC) using the following formulae:

$${Area}_{Trapezoid}=½1/2\;\left(C_{1}+C_{2}\right)\;\left(t_{2}-t_{1}\right)$$

$${Area}_{Trapezoid}=½1/2\;\left(C_{1}+C_{2}\right)\;\Delta t$$

The ratio of AUC of florfenicol amine to the sum of AUC of florfenicol and florfenicol amine was used to calculate the apparent metabolic rate (AMR) of florfenicol amine [AMR = AUC_FFA_/(AUC_FF_ + AUC_FFA_)]. The apparent distribution rate (ADR) of florfenicol amine was calculated as the ratio of florfenicol amine concentration to total florfenicol and florfenicol amine concentrations [*ADR = C*_*FFA*_ */*(C_*FF+*_C_*FFA*_)]. The metabolite ratio (MR) of florfenicol amine was obtained as the ratio of AUCs of florfenicol amine and florfenicol (MR = AUC_FFA_/AUC_FF_). The absorption rate constant (*K*_*a*_) calculated as:

$$K_{a}=\ln\left(2\right)/\;t_{1/2a}$$

Furthermore, PAST 4.04 (Pourmolaie et al., 2018) was used for principal component analysis (PCA) and correlation, using linear R (Pearson) and table format in permutation (P).

### 2.9 Biosafety of florfenicol

#### 2.9.1 Experimental set up

For biosafety of the florfenicol, rainbow trout fingerlings of average length 110.00 ± 021 mm and weight 25 ± 0.015 g were stocked in rectangular FRP tank of 1 ton capacity each. Fish were fed standard feed at the rate of 2% of their body weight twice a day. Feeding behaviour of the experimental fish was observed to be normal during the acclimation phase. The acclimatized fish were separated into 4 treatment groups (1×, 3×, 5× and 10×) and control one in triplicates, 1 week before the experiment. To assess the biosafety, florfenicol at dosage of 10 mg kg^−1^ (1×), 30 mg kg^−1^ (3×), 50 mg kg^−1^ (5×), and 100 mg kg^−1^ (10×) was administered orally through pelleted medicated feed everyday for 30 days. The medicated feed with desired concentration of the antibiotic was consumed immediately by the rainbow trout fingerlings post broadcasting that possibly minimized the drug leaching into the water. After 30 days of antibiotic therapy, the fish were given antibiotic-free diet for another 10 days. Throughout the trial, the control group of fish received pelleted feed without the antibiotic.

### 2.10 Antibiotic feed preparation

Vegetable oil was employed as a binder in the preparation of the medicated feed (de Oliveira et al., 2018). The designated concentrations of florfenicol were measured based on the fish body weight. Then the antibiotic at required concentrations was mixed with 5% vegetable oil. The feed (Nutrila, manufactured by Growel feeds Pvt. Ltd.) according to fish body weight was added to this combination and stirred until the mixture (vegetable oil and antibiotics) was equally coated to the feed. After that the feed was dried in the dark for 1 h before being stored at 4°C for use. A comparable approach was followed to prepare the control feed without adding antibiotic.

### 2.11 Sample collection

The animal behaviour, percentage of feed consumed and survival percentage of the test fish in control and treatment groups were examined on the daily basis during the acclimatisation phase, the antibiotic feeding period and post-antibiotic feeding period, Fish from each tank were randomly collected and weighed on the 10th, 20th, 30th, and 40th day of the experiment. The blood samples were collected from the caudal region and stored in heparinized tubes. A portion of a blood samples was utilised for haematology profiling and the remaining sample was centrifuged at 4,000 x g for 10 min to collect the plasma and kept at −80 C for further analysis. The muscle, liver, kidney, gills, and intestine tissues were taken in Davidson fixative for the study of histological changes. The muscle tissues were subjected to analysis of the antibiotic residues.

### 2.12 Biochemical analysis of blood

A commercial kit was used to test all the plasma parameters. Total protein content was determined using the biuret technique (Flack and Woollen, 1984) and albumin content through BCG dye method (Doumas et al., 1972). The glucose oxidase technique was used to determine the blood glucose level (Trinder, 1969). To calculate the globulin level, the albumin content was subtracted from the total protein.

### 2.13 Plasma immunomodulation

Kinetic technique was used to measure the amount of creatinine with the help of a commercial kit (Jaffe et al., 1980). The activities of aspartate transaminase (AST) and alanine aminotransferase (ALT) enzymes were measured using the technique of International Federation for Clinical Chemistry (IFCC), whereas the activity of alkaline phosphatase (ALP) was approximated using method published earlier (Wilkinson et al., 1969).

### 2.14 Histological examination

The standard method was followed for the histological preparation. In brief, the muscle, liver, kidney, gills, and intestine collected from both treatment and control fish were fixed in Davidson fixative for 18–24 h before being replaced with 70% ethanol. The tissue block was sectioned (4.0, Thermo Scientific Microm HM 323) and stained with hematoxylin and eosin (Bancroft et al., 1996). The dried slides were observed under a light microscope after being mounted with DPX (Dibutylphthalate Polystyrene Xylene) (Olympus IX53, Camera Q-IMAGING, 01-MP 3.3- R- CLR-10, Color RTV10 BIT, Light source OLYMPUS, TH4-200).

### 2.15 Quantification of florfenicol by LC-MS/MS

The chopped and homogenised fish sample (2 g) was weighed into a 50 mL centrifuge tube. Ethyl acetate 10 mL with 2% ammonium hydroxide was used to extract the samples. The sample was vortexed for 2 min to improve analyte extraction. Then, samples were kept in agitation for 20 min followed by centrifugation at 4,000 rpm for 10 min at low temperature (4°C). The supernatants were transferred to a clean tube and evaporated until dried under a gentle flow of nitrogen. Dry residues were subjected to fat removal using 2 mL of hexane, followed by liquid-liquid extraction using 2 mL of water:acetonitrile (50:50,v/v). An aliquot of 1 mL of the bottom layer was transferred to an HPLC vial after soft agitation and analysed in an LC-MS/MS system.

### 2.16 Statistical analysis

At the completion of pharmacokinetic and biosafety study, the one-way analysis of variance (ANOVA) and principal component analysis (PCA) were performed using IBM SPSS (Statistical package for social science) software version 19.0 (SPSS Inc., Chicago IL) to analyze the data. The result was expressed as mean ± SD (standard deviation of the mean) and considered significant at *p* < 0.05. The Student’s t-test was performed to determine the differences between the groups. The comparison was made between treatments and control group to study the significant difference (*p* < 0.05).

## 3 Results

### 3.1 Pharmacokinetic of florfenicol

**FIGURE 1:**
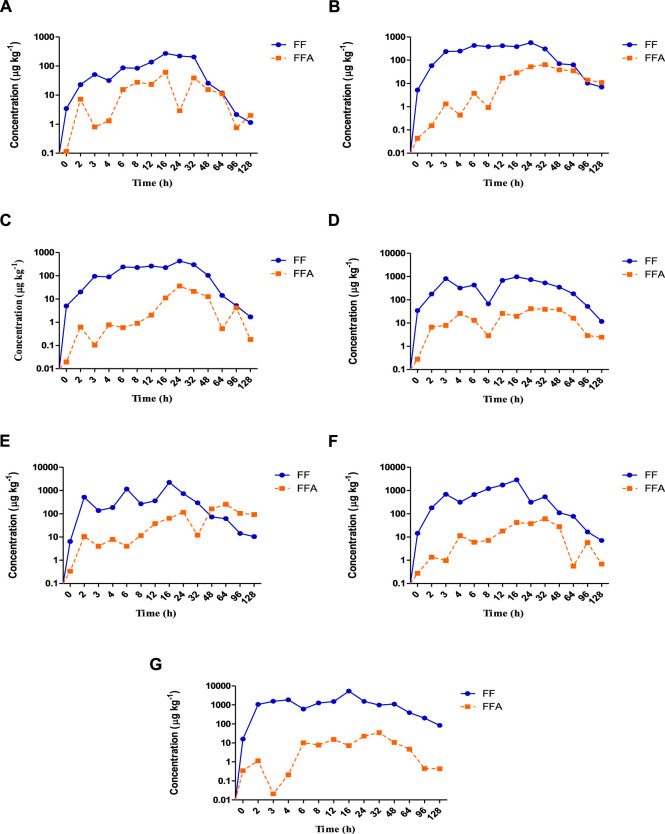
**(A–G)**: The concentration vs. time curves of florfenicol (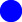) and florfenicol amine (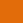) in rainbow trout after a single dose administration of florfenicol at 15 mg kg^−1^ fish body weight. **(A)**: Plasma; **(B)**: Skin; **(C)**: Muscle; **(D)**: Liver; **(E)**: Kidney; **(F)**: Gill; **(G)**: Intestine.

Figures 1A–G shows the concentration–time curves for the florfenicol and its metabolite florfenicol amine in plasma and other tissues.

### 3.2 Distribution

The distribution phase was marked in the Kidney 2 h post oral administration of florfenicol mediated feed, followed by plasma, skin, muscle, liver, or gills at 3 h and intestine at 4 h. The distribution of the antibiotic lasted up to 16 h in all the tissues except muscle and skin. The kidney recorded the lowest concentration of 138 μg kg^−1^ at 3 h, whereas intestine 604 μg kg^−1^ at 6 h during the distribution phase. Other tissues had the lowest concentrations of florfenicol at 4 h. The concentration-time curve showed peaks in plasma, muscle, and other tissues occurred at 6 h. Further, in the drug-time curve, the main peak for intestine occurred at 8 h, and during this stage, the drug’s behaviour prioritised the distribution. The relative distribution level of florfenicol between the plasma and tissues post administration based on the area under concentration-time curve (AUC) values showed intestine had the utmost AUC_(tissue/plasma)_ of 13.83 h μg kg^−1^ followed by gill (5.65 h μg kg^−1^), skin (4.55 h μg kg^−1^), kidney (4.06 h μg kg^−1^), muscle (2.32 h μg kg^−1^), and liver (1.69 h μg kg^−1^) (Table 1). These results showed that florfenicol was widely disseminated throughout all the tissues studied.

**TABLE 1:** Determination of pharmacokinetic parameters of florfenicol post oral administration of medicated feed to rainbow trout at 15 mg Kg^−1^ body weight.

Pharmacokinetic parameter	Florfenicol						
	Plasma	Skin	Muscle	Liver	Kidney	Gill	Intestine
t_1/2a_	23.34	25.13	22.04	20.93	22.70	19.39	28.62
T	33.67	36.26	31.80	30.19	32.75	27.97	41.29
Λ	0.029	0.027	0.03	0.033	0.030	0.035	0.024
*T*_max_(h)	16	24	24	16	16	16	16
*C*_max_ (µg kg^−1^)	273	569	431	973	2,250	2,890	5,360
AUC_(0–128)_	7,735.43	17,964.7	13,076.4	35,202.0	31,468.1	43,779.0	107,043.1
AUC_(tissue/plasma)_		4.55	2.32	1.69	4.06	5.65	13.83
*k*_*el*_	0.03489	0.03143	0.03503	0.02433	0.03334	0.03869	0.022512
AUC_(t-*∞*)_	32.6713	221.099	48.5261	480.814	311.896	183.210	3,722.423
AUC_(0-*∞*)_	7,768.10	18,185.8	13,124.9	35,682.8	31,780.0	43,962.2	110,765.5
K_a_ (h^−1^)	0.030	0.028	0.031	0.033	0.031	0.036	0.024

t_1/2a_: half life, T: decay constant, λ: mean life time, *T*_max_: maximum time, *C*_max_: maximum concentration, AUC (h μg kg^−1^): area under concentration-time curve, *k*_*el*_: elimination constant, K_a_: absorption rate constant.

### 3.3 Absorption

During the absorption phase, the florfenicol concentration in the intestine rapidly increased, reaching to a maximum of 5,360 μg kg^−1^ at 16 h post administration of the antibiotic. The absorption rate decreased after 16 h, and the concentration declined to 83.8 μg kg^−1^ at 128 h. The maximum florfenicol concentration in the skin recorded was 569 μg kg^−1^ at 24 h. The plasma, liver, gill and kidney demonstrated the concentrations of 273 μg kg^−1^, 973 μg kg^−1^, 2,890 μg kg^−1^ and 2,250 μg kg^−1^ fish body weight respectively at 16 h, while the muscle registered a concentration of 431 μg kg^−1^ of florfenicol at 2 h post administration.

### 3.4 Elimination

Other than skin and muscle, the elimination phase appears post 16 h in the plasma, liver, kidney, gill, and intestine. During this profile, the drug level declined rapidly to 1.14 μg kg^−1^ in plasma, 6.95 μg kg^−1^ in skin, 1.7 μg kg^−1^ in muscle, 11.7 μg kg^−1^ in liver, 10.4 μg kg^−1^ in kidney, 7.09 μg kg^−1^ in gill and 83.8 μg kg^−1^ in intestine. The elimination half lives in plasma and other tissues in the increasing order were intestine (28.62 h)>skin (25.13 h)> plasma (23.34 h)> kidney (22.70 h)> muscle (22.04 h)> liver (20.93 h)> gill (19.39 h).

### 3.5 Activity of florfenicol amine

The concentrations of florfenicol amine (the major metabolite of florfenicol) *versus* time in plasma and other tissues after oral florfenicol dosing are also shown in Figures 1A–G and the corresponding pharmacokinetic parameters are stated in Table 2. The maximum time (*T*_max_) for both florfenicol and florfenicol amine to have maximum concentrations of antibiotic in plasma (16 h) and muscle (24 h) were found to be equal. The delayed *T*_max_ (32–64 h) of florfenicol amine with those of florfenicol *T*_max_ (16–24 h) were measured in the skin, liver, kidney, gill and intestine. The maximum concentrations of florfenicol amine (µg kg^−1^) were observed in the order of kidney (258)>skin (64.7)> gill (61.3)> plasma (61)> liver (41.7)> muscle (36.1)> intestine (34.4).

**TABLE 2:** Determination of pharmacokinetic parameter of florfenicol amine post oral administration of medicated feed to rainbow trout at 15 mg Kg^−1^ body weight.

Pharmacokinetic parameter	Florfenicol amine						
	Plasma	Skin	Muscle	Liver	Kidney	Gill	Intestine
t_1/2a_	33.82	50.07	16.75	31.39	86.95	19.74	20.28
T	48.79	72.24	24.17	45.28	125.44	28.48	29.27
Λ	0.020	0.01	0.041	0.02	0.007	0.035	0.034
*T*_max_ (h)	16	32	24	24	64	32	32
*C*_max_ (µg kg^−1^)	61	64.7	36.1	41.7	258	61.3	34.4
AUC_(0–128)_	1,647.46	3,543.68	985.702	2,238.43	15,294.8	2076.76	1,053.239
AUC_(tissue/plasma)_		2.15	0.59	1.35	9.28	1.26	6.38
*k*_*el*_	0.01216	−0.0218	0.00241	0.01099	−0.0241	0.01359	0.007328
AUC_(t-*∞*)_	165.25	1812.719–504.21	74.9184	224.642	−3,858.9	50.3780	59.22719
AUC_(0-*∞*)_	1812.71	3,039.46	1,060.62	2,463.07	11,435.8	2,127.14	1,112.467
MR (%)	0.213	0.197	0.075	0.064	0.486	0.047	0.010
K_a_ (h^−1^)	0.020	0.014	0.041	0.022	0.008	0.035	0.034

t_1/2a_: half life, T: decay constant, λ: Mean life time, *T*_max_: maximum time, *C*_max_: maximum concentration, AUC (h μg kg^−1^): area under concentration-time curve, *k*_*el*_: elimination constant, K_a_: absorption rate constant, MR: metabolite ratio.

Table 2 shows AUC_(tissue/plasma)_ of florfenicol amine. The AUC_(tissue/plasma)_ of florfenicol amine was the highest in the kidney (9.28 h μg kg^−1^) followed by intestine (6.38 h μg kg^−1^), skin (2.15 h μg kg^−1^), liver (1.35 h μg kg^−1^), gill (1.26 h μg kg^−1^) and the lowest concentration was measured in the muscle (0.59 h μg kg^−1^). These results indicated that florfenicol amine was broadly disseminated throughout all tissues. In comparison to florfenicol, the metabolite florfenicol amine was eliminated more slowly in the rainbow trout. The elimination of florfenicol amine was the fastest in muscle with a half-life of 16.75 h. After 48 h, the metabolite concentration was very low (0.527 h μg kg^−1^). Gill had the second fastest elimination; with a half-life of 19.74 h. The rapid elimination was also seen in the intestine, with a half-life of 20.28 h, which registered a metabolite concentration of less than 0.5 μg kg^−1^ post 128 h. The moderate elimination of the antibiotic was observed in the liver and plasma, with half-lives of 31.39 h and 33.82 h, respectively, while a slower elimination rate was observed in the skin and kidney, with half-lives of 50.07 h and 86.95 h, respectively.

### 3.6 Apparent distribution rate (ADR) and the apparent metabolic rate (AMR) of florfenicol amine

Table 3 shows the apparent metabolic rate (AMR) and the apparent distribution rate (ADR) of florfenicol amine in plasma and tissues of rainbow trout post florfenicol oral dosing. The relatively higher AMR was found in the kidney (0.327), moderate in plasma (0.175), skin (0.164) and lower in muscle (0.070), liver (0.059), gill (0.045) and the lowest in the intestine (0.009). The apparent distribution rate (ADR) of florfenicol amine was in the order of plasma (0.129)>kidney (0.049)>liver (0.038)>skin (0.027)>gill (0.024)>muscle (0.022)>intestine (0.006) (Table 3). The ADR in the skin gradually decreased after 64 h post dosing. All ADR values were greater than 0.5 in the kidney from 48 to 128 h and in the plasma at 128 h (Supplementary file: Annexure A).

**TABLE 3:** The apparent metabolic rate (AMR) and the apparent distribution rate (ADR) of florfenicol amine [AMR = AUC_FFA_/(AUC_FF_ + AUC_FFA_); ADR = C_FFA_/(C_FF+_C_FFA_)] in rainbow trout plasma and other tissues post medicated feeding of florfenicol at 15 mg kg^−1^ fish body weight.

Parameter	Florfenicol amine						
	Plasma	Skin	Muscle	Liver	Kidney	Gill	Intestine
AMR	0.175	0.164	0.070	0.059	0.327	0.045	0.009
ADR	0.129	0.027	0.022	0.038	0.049	0.024	0.006

AUC_FFA_, AUC_FF_, C_FFA_, C_FF_.

### 3.7 Principal component analysis (PCA)

**FIGURE 2:**
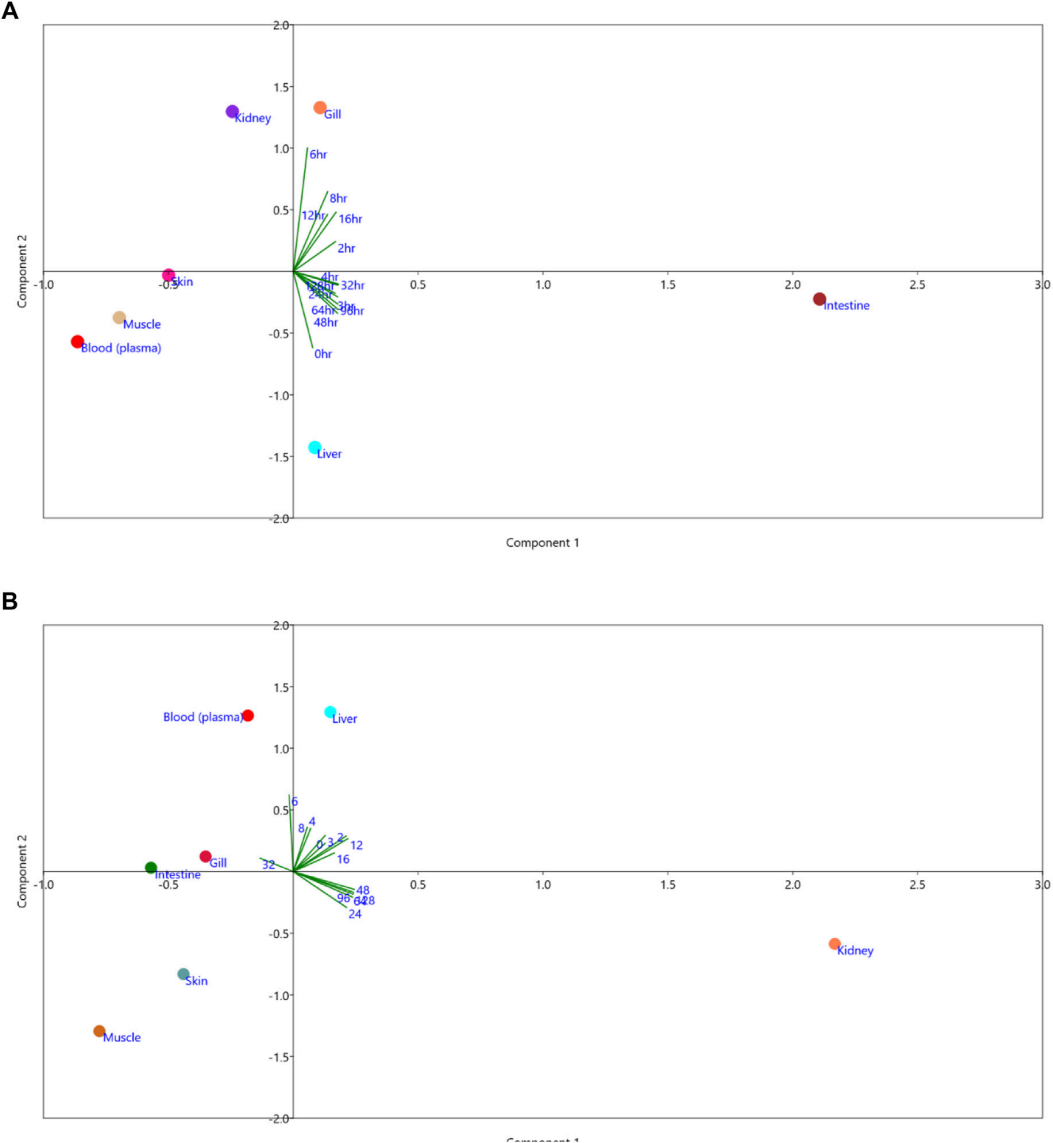
Principal Component Analysis **(A)** Florfenicol **(B)** Florfenicol amine.

The principal component analysis (PCA) showed the minimal consumption of florfenicol in the liver and gill at 0 h and 6 h. At 24–128 h, the highest consumption of florfenicol was observed in the intestine (Figure 2A). The presence of florfenicol amine residues was not found in muscle, skin, intestine, gill, or plasma at any time point during the tissue collection. At 4–8 h, there was less florfenicol amine remainant in the liver. The florfenicol amine residue was found in the highest concentration in the kidney at the 48–128 h time point (Figure 2B).

### 3.8 Pharmacokinetics correlation on permutation basis

Analysis of pharmacokinetic correlation on permutation basis indicated that the concentration of florfenicol at the beginning was lower in each organ. All organs had a favorable link with blood. Because of blood circulation, the florfenicol concentration in the fish body; muscle, and liver has the highest values among other organs (Table 4). For the florfenicol amine, only the kidney exhibited a positive association with time, indicating that at the end of the experiment, the kidney had a concentration of florfenicol amine (Table 5).

**TABLE 4:** Pharmacokinetics correlation on permutation basis of florfenicol.

	Hours	Plasma	Skin	Muscle	Liver	Kidney	Gill	Intestine
Hours		0.2655	0.0625	0.1764	0.1294	0.211	0.1757	0.1745
Plasma	0.2655		0.0004	0.0002	0.0007	0.0042	0.0074	0.003
Skin	0.0625	0.0004		0.0001	0.0062	0.0233	0.0181	0.0543
Muscle	0.1764	0.0002	0.0001		0.0123	0.0765	0.0837	0.1481
Liver	0.1294	0.0007	0.0062	0.0123		0.0079	0.0056	0.0006
Kidney	0.211	0.0042	0.0233	0.0765	0.0079		0.0118	0.0137
Gill	0.1757	0.0074	0.0181	0.0837	0.0056	0.0118		0.0016
Intestine	0.1745	0.003	0.0543	0.1481	0.0006	0.0137	0.0016	

**TABLE 5:** Pharmacokinetics correlation on permutation basis of florfenicol amine.

	Hours	Plasma	Skin	Muscle	Liver	Kidney	Gill
Hours		0.47585	0.45283	0.976	0.61031	0.026692	0.70824
Plasma	−0.20784		0.16443	0.44396	0.31893	0.78189	0.010125
Skin	0.21857	0.39306		0.000282	0.000759	0.081079	0.000243
Muscle	−0.0088658	0.22277	0.82492		0.001263	0.484	0.000581
Liver	−0.14936	0.28749	0.79076	0.77042		0.41115	0.000653
Kidney	0.58895	−0.08147	0.48179	0.2041	0.23871		0.99114
Gill	−0.10996	0.66056	0.82952	0.80063	0.79636	0.003272	

### 3.9 Biosafety of florfenicol

#### 3.9.1 Water quality parameter

Regular monitoring of physical and chemical parameters revealed that the tanks had good quality water similar to pharmacokinetic trial as stated previously. However, the average water temperature for the experimental trial in biosafety experiment was 19.5°C± 0.15°C.

### 3.10 Behaviour and mortality

None of the fish in the experimental groups showed signs of aberrant behaviour such as gasping for air, flashing, hyperactivity, lethargy, loss of equilibrium, or abnormal pigmentation. There was 100% survival in all the treated groups during 30 days of oral antibiotic medication and 20 days of further monitoring except mortality of one fish in the 10× florfenicol group.

### 3.11 Feed intake

Feed intake characteristics were all assigned scores of 0–4. During first 10-day trial, there was no difference in feed consumption of fish between the control and treatment groups. After 10 days, the feed intake by the test fish was dropped with increasing florfenicol treatment period in the 1×, 3×, 5×, and 10× groups. The experimental fish in 10× group demonstrated lowest feed intake (Table 6).

**TABLE 6:** Feed intake in florfenicol-dosed rainbow trout juveniles at 1×, 3×, 5× and 10× of the therapeutic dose 10 mg kg^−1^ biomass day^−1^.

Periods		Florfenicol feeding behavior scoring (mean ± SD)			
Control		Treatment 1× (10 mg kg^−1^)	Treatment 3× (30 mg kg^−1^)	Treatment 5× (50 mg kg^−1^)	Treatment 10× (100 mg kg^−1^)
Pre medicated feeding (0–10 days)	4.0 ± 0.0	4.0 ± 0.0	4.0 ± 0.0	4.0 ± 0.0	4.0 ± 0.0
Florfenicol medicated feeding (11–40 days)	4.0 ± 0.0*	3.0 ± 0.53	3.0 ± 0.52	3.0 ± 0.53	2.0 ± 0.52*
Post medicated feeding (40–50 days)	4.0 ± 0.0*	3.0 ± 0.53	3.0 ± 0.52	2.0 ± 0.53	2.0 ± 0.53*

Feed intake; 1: 25%, 2: 50%, 3: 75%, 4: 100%. *Significance difference at <0.05.

### 3.12 Blood glucose and protein estimation

**FIGURE 3:**
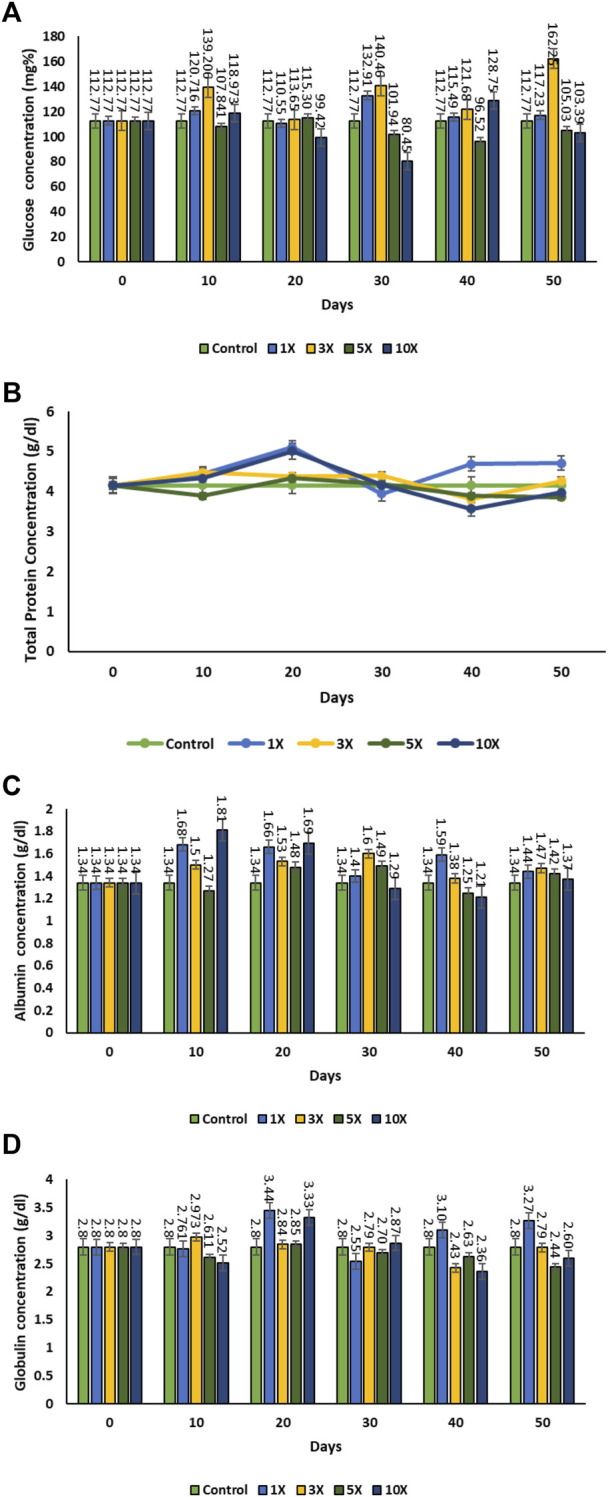
**(A–D)**: Total protein, Albumin, Blood glucose, globulin level of contents in florfenicol-dosed rainbow trout juveniles at 1×, 3×, 5× and 10× times the therapeutic dose of 10 mg kg^−1^ biomass day^−1^ for 30 consecutive days; **(A)** glucose concentration, **(B)** total protein, **(C)** albumin concentration, **(D)** globulin concentration.

The blood glucose levels increased considerably in the 3X concentration of florfenicol with treatment time from an average value of (control) 112.77 ± 0.065 mg dL^−1^ on 0 day, which was correlated with the blood drug concentration (Figures 3A–D). But the blood glucose level was found to be non-significant at higher antibiotic concentrations. Twenty days post antibiotic feeding showed a declining trend in the blood glucose level at 5× and 10× concentrations, which did not recover to the normal value. The total protein concentration was found to be the highest in the 1× fish group ranged during the florfenicol treatment period. Once, the antibiotic the treatment was discontinued post 30 days feeding, the total protein concentrations returned to the near-normal levels in all the treatment groups. However, the levels remained much higher in the 1× group. This might be due to an adaptive physiological response to the medication at low concentrations. The plasma albumin content varied significantly depending on the dose and duration of treatment. It increased noticeably in 1× group, but dropped at higher antibiotic concentrations on the 20th and 30th day of the experimental period. The globulin level declined as the experimental period progressed. The highest increment in globulin level was observed on the 20th day of the experiment in both 1× and 10× groups, which decreased drastically on the next sample. But, following that, the globulin level in the 1× group remained high.

### 3.13 Immunomodulatory effect

**FIGURE 4:**
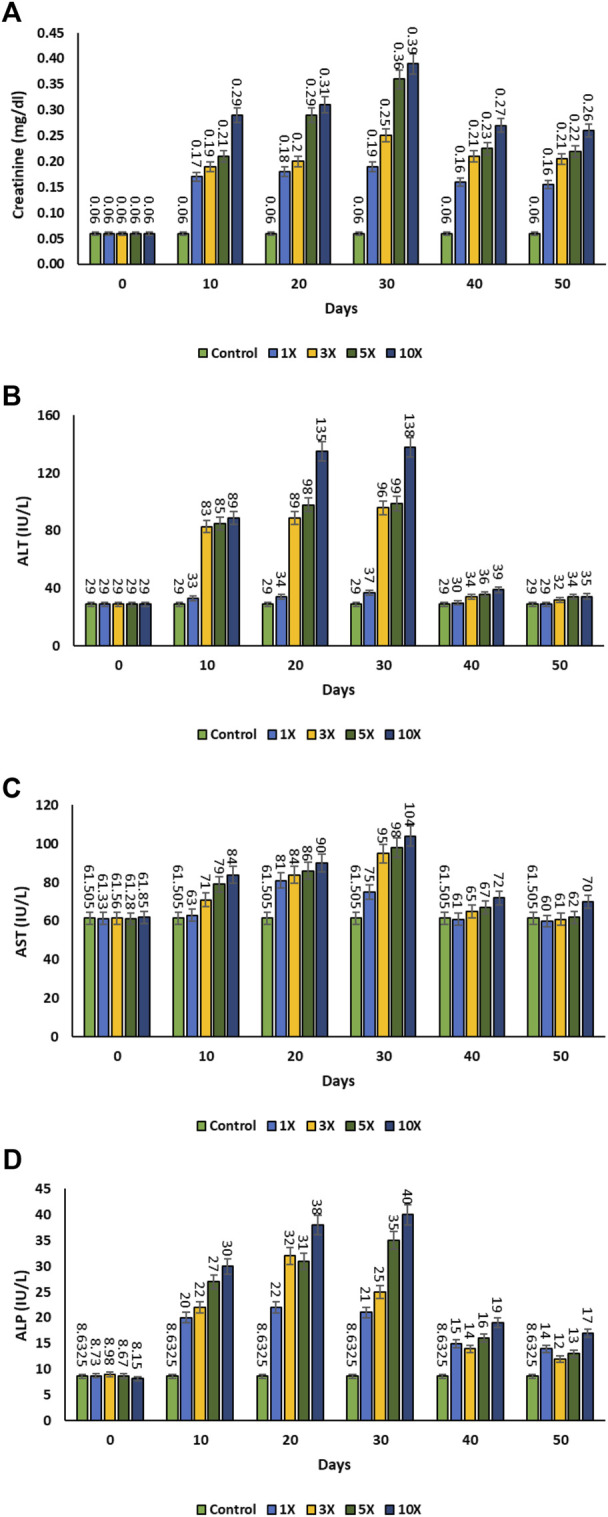
**(A–D)**: Plasma immunomodulatory effect of florfenicol-dosed rainbow trout juveniles at 1×, 3×, 5× and 10× times the therapeutic dose of 10 mg kg^−1^ biomass day^−1^ for 30 consecutive days; **(A)** creatinine, **(B)** Alanine transaminase (ALT), **(C)** Aspartate transaminase (AST), **(D)** Alkaline phosphatase (ALP).

In the control group, the serum creatinine level was found to be 0.06 ± 0.001 mg dL^−1^. The serum creatinine level was increased in all the treatment groups on 30th day and began to decline once the antibiotic dosing was stopped after 30th day. There was no significant variation in creatinine levels between the 1×, 3×, and 5× groups on 40th day post dosing of florfenicol. However, the apparent results showed that the creatinine level was higher at 10× concentration in comparison to other treatment groups (*p* < 0.05) (Figure 4A). The Alanine transaminase (ALT) level in the plasma of the control group was 29.00 ± 1.06 IU L^−1^. The ALT levels in the 1×, 3×, 5×, and 10× groups considerably increased from day 10th to the 30th day, but it decreased following florfenicol post dosing (i.e., after the 30th day). There were no significant changes in ALT levels (*p* > 0.05) in the control, 1×, 3×, 5×, and 10× groups after 30th day of florfenicol administration (Figure 4B). There was significant increase in aspartate transaminase (AST) levels on the 20th and 30th days in 1× group in comparison to 10th day. Then, gradually the AST value decreased 40th day onwards till the end of the experimental period. Similarly, the 3×, 5×, and 10× groups experienced a significant increase in AST levels (*p* < 0.05) till 30th day of florfenicol dosing, after which their levels declined. Except 1× group, the AST levels of all other treatment groups returned to the near normal values on the 40th day and subsequent sampling (Figure 4C). On all days of observation, the alkaline phosphatase (ALP) levels of the 1×, 3×, 5×, and 10× groups were substantially greater than the control (*p* < 0.05). The ALP level in 10× group was significantly high among all the groups and a marked reduction (*p* < 0.05) was observed after 30th day post dosing (Figure 4D).

### 3.14 Histological alterations

**FIGURE 5:**
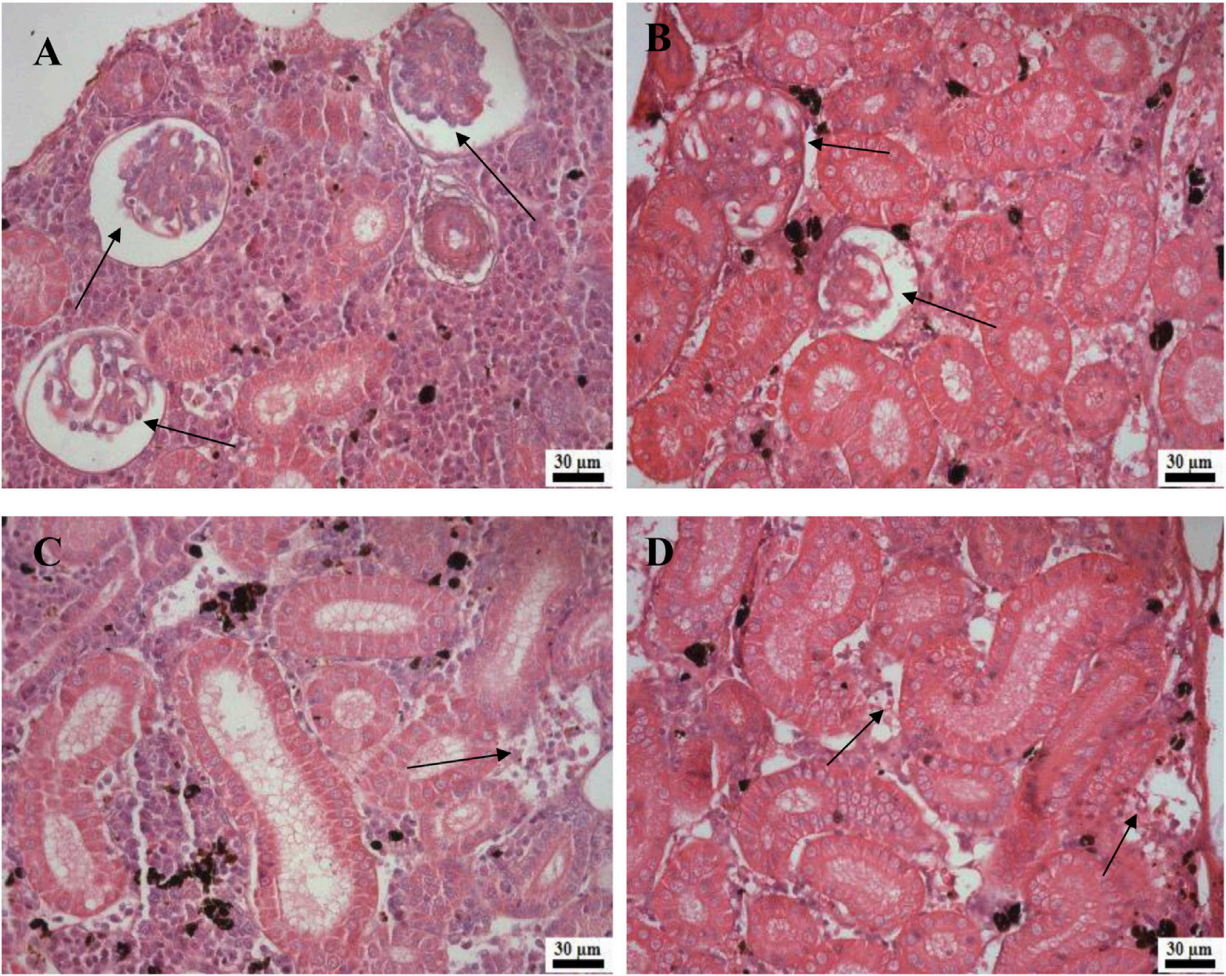
**(A–D)**: Histological changes in rainbow trout kidney post exposure to 5× and 10× florfenicol concentrations on the 30th and 40th day of sampling period; shrunken glomerular tufts and dilation of Bowman’s capsule (arrows) **(A–B)**, periglomerular lymphocytic aggregation (arrow) **(C–D)**.

**FIGURE 6:**
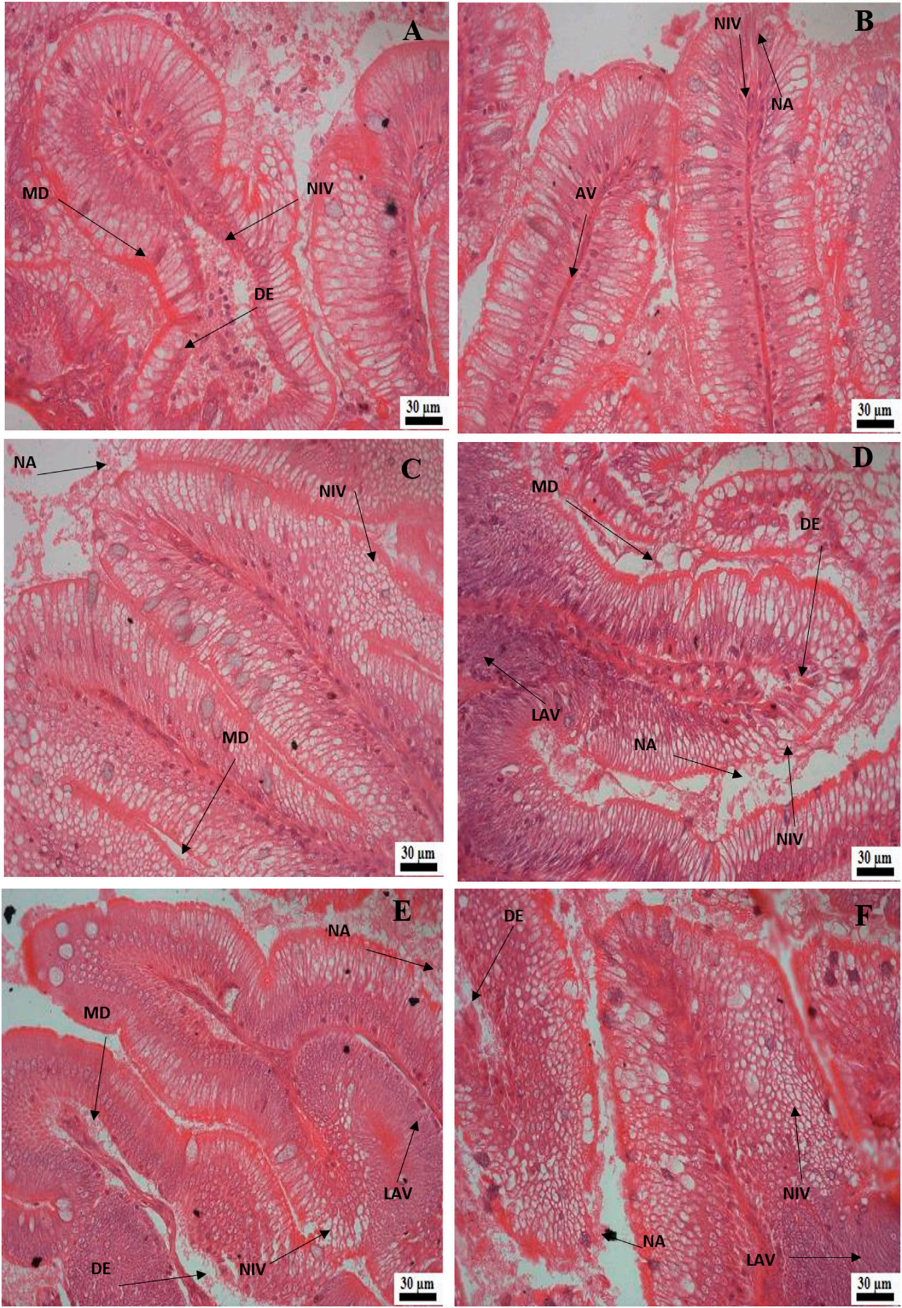
**(A–F)**: Histological alterations in rainbow trout intestine post exposure to florfenicol; **(A)** 30th day at 1× concentration: degenerated epithelial layer (DE), necrosis in the intestinal villi (NIV), mucinous degeneration (MD), **(B)** 30th day at 5× concentration: absorptive vacuole (AV), necrotised absorptive region (NA), necrosis in the intestinal villi (NIV), **(C)** 30th day at 10× concentration: necrosis in the intestinal villi (NIV), mucinous degeneration (MD), necrotised absorptive region (NA), **(D)** 40th day at 1× concentration: degenerated epithelial layer (DE), necrosis in the intestinal villi (NIV), mucinous degeneration (MD), necrotised absorptive region (NA), loss of absorptive vacuole (LAV), **(E)** 40^th^ day at 5× concentration: degenerated epithelial layer (DE), necrosis in the intestinal villi (NIV), necrotised absorptive region (NA), loss of absorptive vacuole (LAV), **(F)** 40^th^ day at 10× concentration: degenerated epithelial layer (DE), necrosis in the intestinal villi (NIV), mucinous degeneration (MD), necrotised absorptive region (NA), loss of absorptive vacuole (LAV).

**FIGURE 7:**
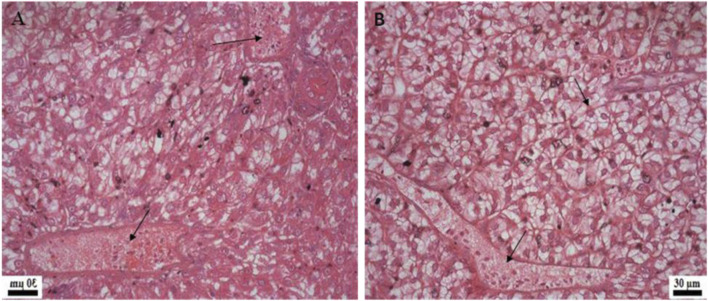
Histological changes in rainbow trout liver post exposure to florfenicol. **(A)** 40th day at 5× concentration: congestion, vacuolar degenerative changes of hepatocytes, **(B)** 40th day at 10× concentration: congestion, extensive vacuolation and degenerative changes of hepatocytes.

Histological examination of the kidney revealed reduced glomerular tufts, dilation of Bowman’s capsule (Figures 5A,B) as well as periglomerular lymphocytic aggregation in the fish groups treated with florfenicol at 5× and 10× concentrations on the 30th and 40th day of sampling period respectively (Figures 5C,D). The intestine recorded degenerated epithelial layer (DE), necrosis in the intestinal villi (NIV), mucinous degeneration (MD), absorptive vacuole (AV), necrotized absorptive region (NA), and loss of absorptive vacuole (LAV) at 1×, 5×, and 10× concentrations on the 30th and 40th day of samplings (Figures 6A–F). Further, due to extended feeding of florfenicol at 5× and 10× concentrations, the liver showed hyperplasia with distorted cell architecture, enhanced vascularisation, and vacuolating changes of hepatocytes, as well as congestion (Figures 7A,B).

### 3.15 Antibiotic residue in muscle

**FIGURE 8:**
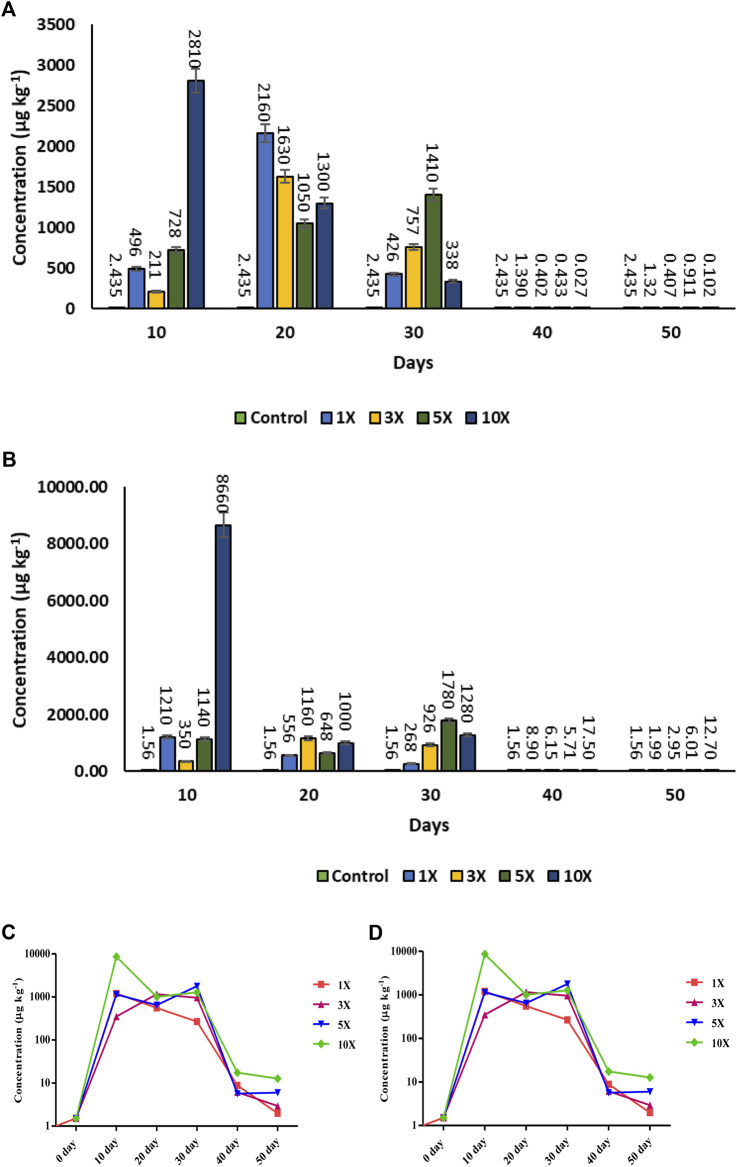
**(A–D)**: Antibiotic residue Concentration of florfenicol (FF) florfenicol amine (FFA) in muscle tissue (µg kg^−1^) in florfenicol dosed rainbow trout juveniles at 1×, 3×, 5× and 10× times the therapeutic dose of 10 mg kg^−1^ biomass day^−1^ for 30 consecutive days; **(A)** Florfenicol bar graph, **(B)** Florfenicol amine bar graph, **(C)** Florfenicol logarithmic scale graph, **(D)** Florfenicol amine logarithmic scale graph) at different time period.

Florfenicol quantification revealed the drug’s greatest prevalence at various times. The length of the dosage administered was substantially linked with the muscle residue concentration of florfenicol (*p* < 0.05). On the 20th day (*T*_max_) of tissue residue, the antibiotic concentration (*C*_max_) was 2,160 μg kg^−1^ and 1,630 μg kg^−1^ in 1× and 3× groups respectively. On the 30th day, the highest concentration of 1,410 μg kg^−1^ was attained in muscle of 5× treated fish. The maximum concentration of 2,810 μg kg^−1^ in muscle tissue of 10× group was obtained on the 10th day of the antibiotic treatment. After that the concentration of antibiotic residue was dropped to 1.32, 0.407, 0.911, and 0.102 μg kg^−1^ in concentrations of 1×, 3×, 5×, and 10×, on 50th day respectively (Figures 8A–C).

In case of florfenicol amine, the *C*_max_ 8,660 and 1,210 μg kg^−1^ were recorded at 10× and 1× concentrations on the 10th day (*T*_max_) of sampling, respectively, whereas at 3× and 5×, the residue concentrations were 350 μg kg^−1^ and 1,140 μg kg^−1^ respectively. On the 50th day, the metabolite concentrations recorded were 1.99, 2.95, 6.01, and 12.7 μg kg^−1^ at 1×, 3×, 5×, and 10× concentrations respectively (Figures 8B–D).

## 4 Discussion

The use of pharmacokinetic and pharmacodynamic characteristics to optimise antimicrobial treatment is a rational and productive approach toward the use of appropriate antimicrobial agent in a clinical environment. The effects of various microbial agents, particularly those that affect important organs involved in drug pharmacokinetic behaviour, is quite limited in veterinary species, particularly aquatic animals (Shiry et al., 2019). Several researches on the pharmacokinetics of florfenicol in various fish species have been conducted. In fish, oral administration is a communal route of medication delivery (Horsberg et al., 1994; Yanong et al., 2005). In the present study, florfenicol was readily absorbed in the gut at a concentration of 5,360 μg kg^−1^ in rainbow trout following an oral dose of 15 mg kg^−1^ treated feed at 17.0°C ± 0.34°C. In the intestine, liver, kidney, gills, and plasma, the time to achieve maximum concentration (*T*_max_) is 16 h. *T*_max_ occurred at 24 h in the muscle and skin, which represented the deep peripheral compartment. Similarly, in crucian carp at a water temperature of 25°C ± 1 C following p. o. Administration at a greater dosage of 40 mg kg^−1^, faster *T*_max_ (0.5 h) was found in liver, kidney, and gill than that of muscle (3 h) (Sun et al., 2010). The pharmacokinetics of a single oral dosage of florfenicol at 10 mg kg^−1^ in plasma was studied in Atlantic salmon in two reasonably similar trials, where *T*_max_ was reported at 6 and 10.3 h (Martinsen et al., 1993; Horsberg et al., 1996). Interestingly, *T*_max_ of orally delivered florfenicol to common carps was detected as early as 2 h after drug administration (Nejad et al., 2017). That indicates that the florfenicol takes less time to reach peak plasma concentration in common carps than salmonids.

The significant re-absorption in the gut and gills was observed at 16 h in the experiment and this developed a next drug peak in the liver after 12 h. Similarly, the peak for plasma, skin, muscle, and kidney occurred at 6 h in the plasma and tissue-concentrations time curve. This developed due to the liver first pass effect and the speedy excretion of plasma, skin, muscle, and kidney during the drug distribution phase. The post oral administration of the other compounds demonstrated the double and triple peaks in the drug-time curve occurred in rainbow trout (Bjorklund and Bylund 1990; Bjorklund, et al., 1992), Atlantic salmon (Elema et al., 1995), and sea bass (Intorre et al., 2000). Multiple peak phenomena have also been observed in plasma and tissues of tilapia (Feng and Jia 2009) as well as in kidney, gill, and bile of crucian carp (Sun et al., 2010). The reabsorption phenomena were recorded in the liver, kidney, gills, gut, plasma, and bile of orange-spotted grouper (Feng et al., 2016). Many peaks were also found in the florfenicol concentrations *versus* time graph in crucian carp only at water temperature of 10 C (Yang et al., 2019). The plasma *T*_max_ of 16 h has been reported earlier in other fish species ranging from 1.5 h in crucian carp (Zhao et al., 2011) to 3 h in koi carp (Yanong et al., 2005), 4 h in olive founder (Lim et al., 2010) and grouper (Feng et al., 2016) and 24 h in gourami (Yanong et al., 2005). At higher water temperature, the *T*_max_ was reported to be shorter (Bjorklund and Bylund 1990; Bjorklund et al., 1992). A longer *T*_max_ of 9 h at 8 C in cod was recorded following oral dosing at a comparable dose of 20 mg kg^−1^ (Samuelsen et al., 2003). This may be attributed to the lower water temperature. Concurring to the above findings, a longer *T*_max_ of 10.3 h in Atlantic salmon reared in sea water at 10.8°C ± 1.5°C can also be attributed to the lower water temperature. Similarly, when fish were fed with a diet, where florfenicol was coated exteriorly, resulted in a shorter *T*_max_ of 6 h (Horsberg et al., 1996). In comparison to the saltwater fish, the freshwater tilapia showed a longer *T*_max_ of 12 h following administration with the pellet dosage form at 22 C (Feng and Jia 2009).

The highest concentrations (*C*_max_) of florfenicol was found in the intestine (5,360 μg kg^−1^) followed by other tissues, was also in accordance to the previous findings in grouper (Feng et al., 2016), whereas, the *C*_max_ in the cod was determined at 10.8 μg mL^−1^ in plasma, 13.0 μg g^−1^in muscle, and 12.1 μg g^−1^in liver (Samuelsen et al., 2003). A decreased *C*_max_ of 0.228, 0.229, and 0.234 μg kg mL^−1^ was found in the crucian carp plasma at varied temperatures of 10, 20, and 30 C respectively (Yang et al., 2019), 0.72 μg kg mL^−1^ mg^−1^ in koi carp (Yanong et al., 2005), 0.71 μg kg mL^−1^ mg^−1^ in olive flounder (Lim et al., 2011), 0.56 μg kg mL^−1^ mg^−1^ in threespot gourami (Yanong et al., 2005), and 0.42 μg kg mL^−1^ mg^−1^ in crucian carp (Zhao et al., 2011). The *C*_max_ was 9.1 μg mL^−1^ in Atlantic salmon following coated pellet feed administration (Horsberg et al., 1996), 4.0 μg mL^−1^ in Atlantic salmon (Martinsen et al., 1993), and 4.46 μg mL^−1^ in tilapia after pellet administration of florfenicol at the dose of 10 mg kg^−1^ (Feng and Jia, 2009).

A half-lives (*t*_*1/*2_) of 36, 16, 11, 11 and 6 h were recorded in the bile, gill, kidney, muscle and liver respectively in case of crucian carp (Sun et al., 2010). In grouper, the elimination half-lives were as follows; bile 13.92 h, muscle and liver 12.31 h, skin 11.77 h and plasma 11.57 h and gill 11.04 h (Feng et al., 2016). When compared to the Atlantic salmon (*t*_*1/*2_ of 14.7 and 12.2 h, t = 10 C), the lumpfish showed elimination *t*_*1/2*_ of 30 h at 12 C, which is described as sluggish (Martinsen et al., 1993; Horsberg et al., 1996). Florfenicol was removed at a slower rate in both Atlantic cod and olive flounder, with *t*_*1/2*_ values of 39 h (t = 8 C) and 49 h (t = 18.5°C), respectively (Samuelsen et al., 2003; Lim et al., 2010). There is a resemblance in elimination between plasma and tissues in lumpfish, ranging from 24 h in muscle to 33 h in head kidney. In Atlantic cod, however, the difference in *t*_*1/2*_ values between plasma (*t*_*1/2*_ = 39 h), muscle (*t*_*1/2*_ = 21 h), and liver (*t*_*1/2*_ = 20 h) varied significantly (Kverme et al., 2019). Specifically, the florfenicol removal was faster in the saltwater fish than in freshwater fish (Feng et al., 2008). Furthermore, it was well established that the exposure temperature had a significant impact on the drug’s clearance rate (Bjorklund and Bylund 1990; Bjorklund et al., 1992). So, it is not unexpected find substantially longer elimination phase *T*_*1/2b*_ values of 12.2 and 14.7 h in cold-water Atlantic salmon following intravenous (i.v) treatment (Martinsen et al., 1993; Horsberg et al., 1996). *T*_*1/2b*_ values of 43 and 39 h were obtained in the cod following i. v. injection and p. o. Administration, respectively (Samuelsen et al., 2003), which was attributed mostly to the lack of a metabolic pathway in cod. *T*_*1/2b*_ values of 38.06 and 51.18 h were recorded in olive flounder kept in sea water at 18.5°C ± 1.7°C following i. v. And p. o. Administration respectively (Lim et al., 2010). At such high-water temperatures, the lack of a metabolic route may only account for a portion of the prolonged *T*_*1/2b*_. In the present study, a prolonged half life of florfenicol in different rainbow trout tissues (intestine 28.62 h, skin 25.13 h, plasma 23.34 h, kidney 22.70 h, liver 20.93 h and gill 19.39 h) was recorded at water temperature of 17.0°C.

The area under concentration *versus* time curve from time zero, extrapolated to 128 h (*AUC*_*0-128h*_) (h μg kg^−1^) of plasma 7,735.43 and other tissues like skin 17,964.7, muscle 13,076.4, liver 35,202, kidney 31,468.1, gill 43,779 and intestine 107,043.1 were calculated in the study. The AUC of Atlantic cod was determined to be 524 h g/mL, which is double that of lumpfish (Samuelsen et al., 2003). This discrepancy is attributable to differences in elimination rates across species. Previous studies only reported AUC values, and when compared to Atlantic salmon, which had AUCs of 140 and 112 h g mL^−1^, respectively (Martinsen et al., 1993; Horsberg et al., 1996), the AUC of lumpfish was roughly twice as large. In plasma, AUC and AUC_0–24h_ were determined to be 248 and 61 h g mL^−1^, respectively (Kverme et al., 2019).

Florfenicol amine, a major metabolite of florfenicol in Atlantic salmon, was measured in greater amounts than florfenicol in plasma after 48 h of the initial injection in a multiple-dose trial (10 mg/kg/day for 10 consecutive days) (Horsberg et al., 1996). The kinetic traits of florfenicol amine were also monitored in the present study and found that most tissues had postponed *T*_max_ at 24–64 h as compared with the *T*_max_ of florfenicol. In case of florfenicol amine, the recorded synchronous *T*_max_ for plasma (16 h) and muscle (24 h) were found in similar to florfenicol. In case of freshwater eel (*Monopterus albus*) and Korean catfish (*Silurus asotus*), the florfenicol and florfenicol amine ratios at *T*_max_ were 4:1 and 3:1 respectively, following a single oral dosing of florfenicol at 20 mg kg^−1^ (Park et al., 2006; Xie et al., 2013). Similarly, delayed *T*_max_ was reported in plasma and other tissues (4–48 h), sooner in bile (6 h), and skin (6 h) (Feng et al., 2016). After administration, the ADR of florfenicol amine were reduced in all tissues except the skin. The maximum values in plasma and kidney demonstrated florfenicol amine’s substantially greater ADR in metabolic and excretory organs when compared to muscle and intestine. The ADR value of plasma (less than 0.5 at 48 h), kidney (0. 69 at 48 h) and skin (0.9) indicated distribution priority of parent drug (Florfenicol) throughout observation period. The distribution level of florfenicol amine in muscle was greater than that of florfenicol post 48–128 h of the treatment. This indicates that the medication is mostly eliminated using florfenicol amine during the terminal elimination phase (Feng et al., 2016) and the metabolite may act as the primary and marker residue for florfenicol. Except for the muscle and gut, where florfenicol amine elimination was relatively rapid than that of florfenicol with T_1/2_ of 16.75 and 20.28 h, respectively. In grouper, an early elimination of T_1/2_ value of florfenicol amine in muscle and liver with values of 16.66 and 17.37 h, respectively was reported (Feng et al., 2016). Contrary to the above finding, a prolonged T_1/2_ in muscle and liver with values of 49 and 56 h was recorded (Horsberg et al., 1996). This could be due to the administration of florfenicol at two different water temperatures (Feng et al., 2016). The elimination half-life in plasma was 33.82 h, which was lower than the 165.04 h in grouper (Feng et al., 2016), but higher than the 23 h and 24.2 h in Atlantic salmon (Horsberg et al., 1996), and 14.03 h and 21.72 h in Korean catfish, respectively (Park et al., 2006). In the skin and kidneys, florfenicol amine was eliminated more slowly (50.07 h and 86.95 h respectively). The affinity of this metabolite for macromolecules, such as melanin, may explain the delayed elimination (Horsberg et al., 1996). In trout plasma, the apparent metabolic rate (AMR) of florfenicol amine was 0.175, whereas, the AMR value in plasma of grouper was 0.23 (Feng et al., 2016), 0.21 and 0.22 in Korean catfish after intravenous and oral administration respectively (Park et al., 2006). This demonstrates that the AMR value recorded in the plasma of rainbow trout in the present study was lower than the earlier reports. Moreover, the higher AMR value of 0.327 found in the kidney of the trout indicated a high level of biotransformation from florfenicol-to-florfenicol amine. The lower AMR values in the other tissues could be due to a difference in affinity between the drug and its metabolite for macromolecules, resulting in AMR differences between tissues (Feng et al., 2016).

The biosafety of florfenicol was carried out at the optimal growth temperature of 19.5°C ± 0.51°C, because drug metabolism is temperature dependant (Bardhan et al., 2022). The feed consumption by rainbow trout, dosed with florfenicol, considerably decreased in a dose-dependent manner, especially in the 3×, 5×, and 10× groups. During the dosing time, the fish ingested the majority of the feed administered and frequently breaching the surface of the water while dining (Bowker et al., 2013; Gaikowski et al., 2013).

The amount of blood glucose is a measure of metabolic rate, and it rises in response to stress. Florfenicol dosage for 30-day significantly elevated glucose levels in all the treatment groups, showing that florfenicol produced stress and changed carbohydrate metabolism (Sopinka et al., 2016; Julinta et al., 2019). The blood glucose levels at the 3× dosage grew considerably over time in the current research, showing the body’s survival reaction to the stress. Plasma albumin levels varied with the dosages and treatment duration, increasing at lower doses in the beginning of therapy and decreasing at higher doses in the later days of the treatment. Because plasma albumin is generated in the liver, the drop has been linked to hepato-pathology and ALT activity, suggesting that low albumin levels at higher dosages of the medication are due to reduced liver function. The highest concentration of globulin was found at 1× concentration, showing a biphasic response with an initial increase and then followed by a significant reduction in the levels. Increased globulin levels at lower concentrations of the drug were thought to be the host’s defence responses to control infection because the globulin fraction has a protective function (Manna et al., 2021).

The considerable increase in ALT levels in the 1× group after 30 and 20 days of florfenicol dosage suggested that florfenicol caused liver tissue deterioration or injury. Nevertheless, in comparison to the control, the increase seen on day 10 of florfenicol dosing at the therapeutic dose (1×) was not significant, suggesting slight injury in the liver (Reda et al., 2013). The dose-dependent elevations in ALT levels as observed in the 3×–10× groups on 10th, 20th and 30th day after florfenicol treatment, indicated florfenicol hepatotoxicity as dose and dosing time increased. On 30th day of florfenicol dosing, the 10× group showed a fivefold rise in ALT levels. Hepatic necrosis and cirrhosis, viral or toxic hepatitis, and obstructive jaundice are all linked to elevated ALT levels (Kim et al., 2008). The ALT levels were more or less recovered on 40th day post-florfenicol dosing. On day 10 of florfenicol dosing, a rise in ALP levels at the therapeutic dose (1×) suggested the likelihood of florfenicol-induced liver inflammation and hepatotoxicity (Labarrere et al., 2013; Soltanian et al., 2018). On day 30 of florfenicol treatment, the ALP levels increased in a dose-dependent manner, with the highest values in the 10× group. These findings are consistent with fish during florfenicol use (Shiry et al., 2019). Despite the fact that the ALP levels in all the treatment groups decreased when florfenicol medication was stopped, they remained considerably high as compared to the day zero. These findings revealed that florfenicol-treated fish was having persistent liver inflammation. The aspartate transaminase (AST) enzyme was found in large quantity in the liver, heart, gill, kidneys, flesh, and other organs, and its presence at greater levels in the blood indicates the proper functioning of these organs (Manna et al., 2021). On 30th day of florfenicol dosing, all the treatment groups had a substantial rise in AST levels, the highest being in the 10× group. A significant rise in AST level confirms hepatotoxicity of florfenicol (Bardhan et al., 2022). Amphenicols and oxytetracycline have been shown to be hepatotoxic (Memik 1975; Julinta et al., 2019). An increase in AST levels was also recorded in *O. mykiss* after using florfenicol (Er and Dik 2014). Drugs or stress can produce changes in serum ALT and AST values that indicate damage of liver tissue (Julinta et al., 2019; Bojarski et al., 2020). The increased AST activity usually indicates acute, rather than chronic, liver necrosis (Kim et al., 2008).

All of the treatment groups had a substantial rise in creatinine levels. Increased serum creatinine levels indicated kidney injury and a decrease or loss of renal function (Julinta et al., 2019). On day 30th of florfenicol treatment, the creatinine levels in the 10× group increased, indicating florfenicol nephrotoxicity (Bardhan et al., 2022). Although in all the treatment groups, the creatinine levels were lower on 40th and 50th day after receiving florfenicol, they were still considerably high. The higher levels of a clinical parameter are frequently interpreted as indicative of organ damage and such an increase within normal or acceptable limits might alternatively indicate the body’s adaptive reaction to changes in bodily function (Manna et al., 2021).

In histological alterations, the previous study showed florfenicol administration at doses of 10, 30, or 50 mg kg^−1^ fish body weight day^−1^ for 20-day in channel catfish resulted in a dose-dependent decrease in hematopoietic/lymphatic tissue in the anterior kidney, posterior kidney and spleen (Gaikowski et al., 2003). Similarly, the florfenicol administration in the feed of tilapia at 15, 45, or 75 mg kg^−1^ body weight for 20-day resulted in increased glycogen and lipid-type in the liver, increased blast cells, and individual cell necrosis in the anterior kidney, and tubular epithelial degeneration and mineralization in the posterior kidney (Gaikowski et al., 2013). In contrary to above findings, it was also reported that florfenicol administration in feed at 75 mg kg^−1^ body weight day^−1^ to sunshine bass for 20-day did not show hepatic tissue alteration (Straus et al., 2012). But, in the present study, the histological changes in both the liver and kidney very much supported the significant changes in enzymatic activities like AST and ALT in blood, indicating tissue impairment due to stress, toxicity, and liver damage caused at 5 × (50 mg kg^−1^ fish body weight) and 10× (100 mg kg^−1^ fish body weight) treatments.

A maximum residual limit (MRL) level of 1,000 μg kg^−1^ body weight in muscle of the fish ingesting florfenicol at 1,000 μg kg^−1^ body weight is recommended by the European Medicines Agency (2000). Unaccounted for leaching losses in water from treated feed may lead to low drug bioavailability and tissue concentrations. However, the quick eating habits of the test fish species and the use of floating feed would limit drug leaching (Xu and Rogers, 1994). Despite the fact that drug concentrations in muscle were low at 1× concentration, throughout the dosing period, the high levels were recorded with 3×−10× dosing. The fast drug transport from blood to muscle might be the reason behind rising levels of the florfenicol (Manna et al., 2021). The concentration of florfenicol amine was low in all the treatment groups compared to the florfenicol metabolism except in 10× concentration of 10th day i.e., 8,660 μg kg^−1^body weight during dosing period and reduced to minimal concentration of 1.5–17.5 μg kg^−1^ body weight once the dosing period was over i.e., on 40th and 50th day. When the fish was treated with the necessary amount of florfenicol, the presence of the antibiotic was significantly below the MRL values, indicating the safety of fish muscle for the human consumption (Manna et al., 2021).

## 5 Conclusion

Florfenicol possesses various desirable antibacterial characteristics; broad spectrum of action, high tissue penetration and minimal resistance. Because of its higher bioavailability, better dispersion, and quicker withdrawal time, the florfenicol and its metabolite are considered a superior antibacterial agent in rainbow trout farming. The pharmacokinetic profile of florfenicol amine can give important information on its relative depletion pattern that may vary from species to species in fish corresponding to the rearing water temperature.

The pharmacokinetic characteristics of florfenicol and florfenicol amine after a single dose oral administration (15 mg kg^−1^ body weight) in rainbow trout, recorded easy absorption of florfenicol in the gastrointestinal tract that distributed to the kidney and other tissues. As a result of the high absorption level of florfenicol in the fish body, the less amount of the antibiotic is discharged into the environment. The antibiotic and its metabolite are removed early from the plasma and other organs than in the skin and muscles. A slow drug excretion may result in a longer drug withdrawal period as compared to ingestion, which is a beneficial therapeutic characteristic. Within 24 h, the drug’s (florfenicol) lowest residual level was obtained in all tissues. In trout, the florfenicol amine is removed mostly by muscle and gill excretion.

In biosafety experiment, the rainbow trout demonstrated higher drug palatability and proper adaptive physiological response to overcome the stress at low dosing of florfenicol (10 mg kg^−1^ fish body weight). The results of clinical parameters indicated non-pathological conditions of liver in 1× group. The histological alterations showed non-toxic conditions of internal organs at low antibiotic dosage 10 mg kg^−1^ fish body weight. Moreover, the antibiotic residue levels in the muscle registered its presence below the MRL limit. It is also established that an extended feeding (or treatment) of florfenicol (30-day) through medicated feed (against recommended 10-day feeding trial), need not necessarily exerted any toxic impact in rainbow trout at low concentration. Though, the considerable toxicity on feed intake, physiological responses and histological alterations in internal organs have been observed at higher concentrations of florfenicol (50 and 100 mg kg^−1^ fish body weight) administered, no mortality of rainbow trout is recorded in the experimental tanks. Thus, feed mediated administration of florfenicol indicates tolerance of the rainbow trout to the drug at varied concentrations (3–10 times of therapeutic dosage). Based on the above findings, the application of florfenicol in rainbow trout is recommended at 10 mg kg^−1^ fish body weight.

## Data Availability

The original contributions presented in the study are included in the article/Supplementary Material, further inquiries can be directed to the corresponding authors.
