# Supplementary material for: Pharmacokinetics and biosafety evaluation of a veterinary drug florfenicol in rainbow trout, Oncorhynchus mykiss (Walbaum 1792) as a model cultivable fish species in temperate water
Source: Front Pharmacol. 2023 Jan 23;14:1033170. doi: 10.3389/fphar.2023.1033170 (PMC9900004; doi:10.3389/fphar.2023.1033170)
Supplement: Supplementary file 1 [file DataSheet1.docx]

Supplementary File: Annexure A

Table: The apparent distribution rate (ADR) of florfenicol amine {*ADR = C_FFA_ /*(C*_FF+_*C*_FFA_*)} in plasma and other tissues of rainbow trout after florfenicol administered through feed at 15 mg kg^-1^ body weight

| Hour | Tissues | | | | | | |
| --- | --- | --- | --- | --- | --- | --- | --- |
| 2 | Plasma | Skin | Muscle | Liver | Kidney | Gill | Intestine |
|  | 0.239 | 0.966 | 0.030 | 0.036 | 0.019 | 0.007 | 0.001 |
| 3 | 0.015 | 0.987 | 0.001 | 0.009 | 0.028 | 0.001 | * |
| 4 | 0.039 | 0.984 | 0.008 | 0.074 | 0.040 | 0.035 | 0.0001 |
| 6 | 0.151 | 0.986 | 0.002 | 0.029 | 0.003 | 0.008 | 0.016 |
| 8 | 0.247 | 0.979 | 0.003 | 0.040 | 0.041 | 0.005 | 0.006 |
| 12 | 0.146 | 0.972 | 0.007 | 0.036 | 0.094 | 0.010 | 0.009 |
| 16 | 0.182 | 0.96 | 0.047 | 0.019 | 0.027 | 0.014 | 0.001 |
| 24 | 0.012 | 0.959 | 0.077 | 0.053 | 0.136 | 0.106 | 0.014 |
| 32 | 0.159 | 0.905 | 0.065 | 0.067 | 0.039 | 0.102 | 0.033 |
| 48 | 0.375 | 0.594 | 0.108 | 0.097 | 0.690 | 0.200 | 0.009 |
| 64 | 0.484 | 0.497 | 0.035 | 0.081 | 0.806 | 0.007 | 0.011 |
| 96 | 0.260 | 0.096 | 0.449 | 0.052 | 0.879 | 0.257 | 0.002 |
| 128 | 0.638 | 0.051 | 0.096 | 0.174 | 0.899 | 0.088 | 0.005 |

* The vacant value, due to the undetectable concentration of either florfenicol or florfenicol amine

Supplementary File: Annexure B


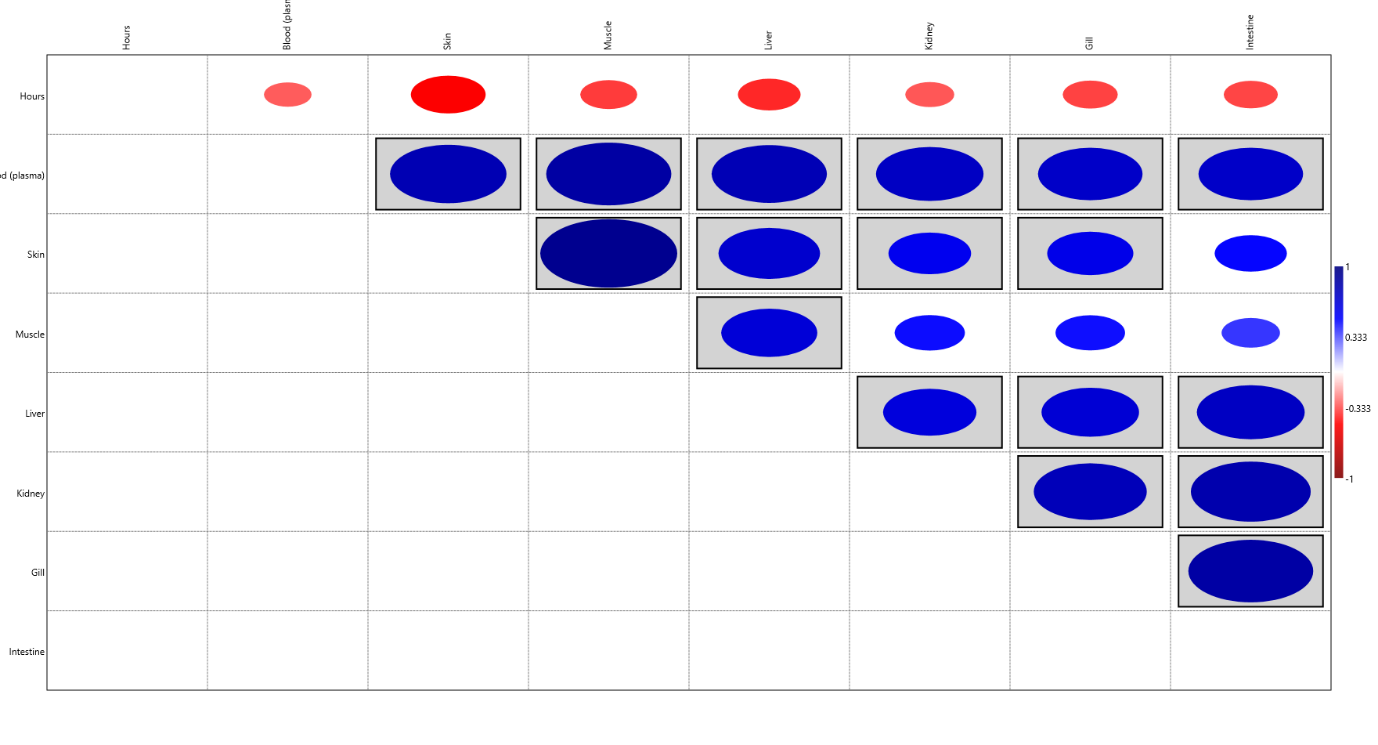


**B**

**A**


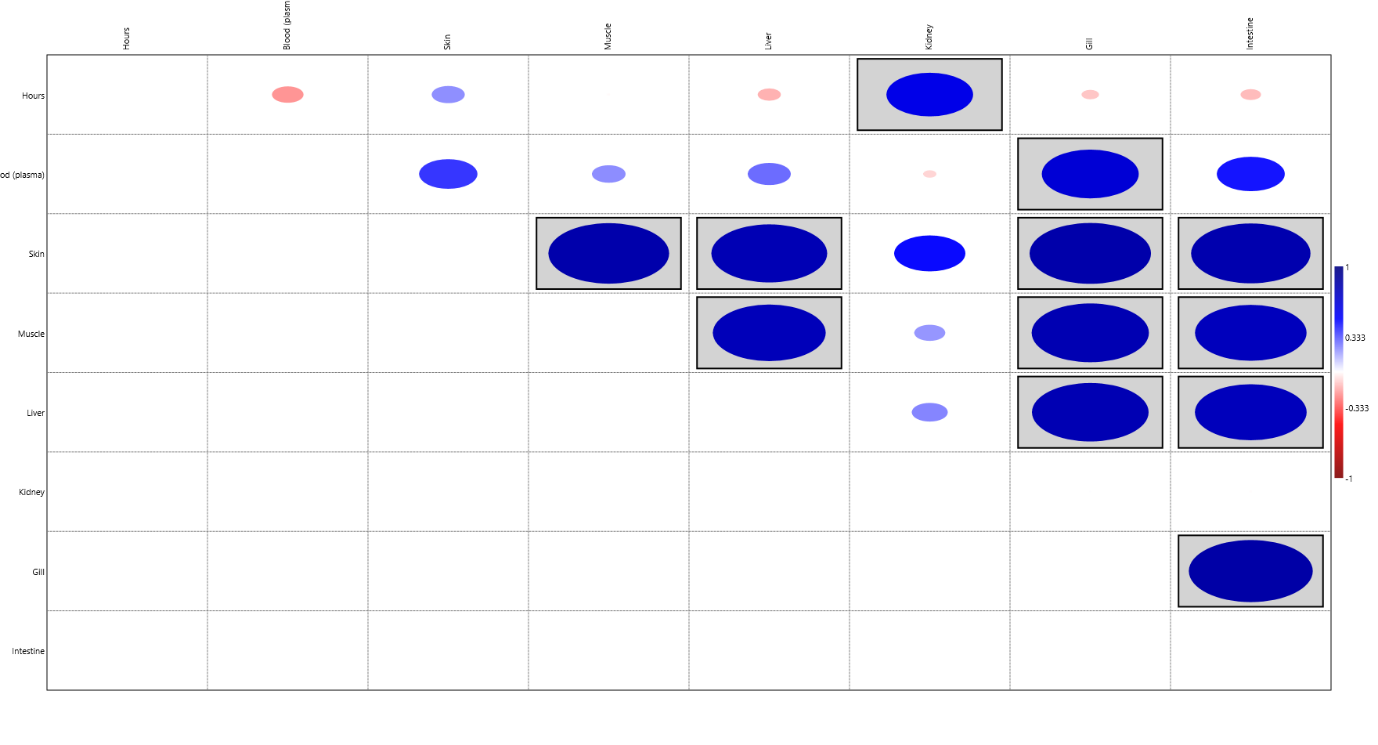


Figure A-B: Pharmacokinetics correlation on permutation basis, (A) florfenicol (B) florfenicol amine. Permutation correlation graph displaying positive and negative relationships between various organs. On the basis of permutation, blue oval shapes indicate a positive association to the corresponding organ, red oval shapes indicate a negative correlation, and boxed blue forms imply 0.333 times positive correlation
